# Operational Challenges in Conducting a Subnational TB Prevalence Survey in India: Lessons Learned for Resource-Limited, High-Burden Settings

**DOI:** 10.9745/GHSP-D-23-00284

**Published:** 2024-02-28

**Authors:** Prathiksha Giridharan, Havenesh Murugesan, Sriram Selvaraju, Asha Frederick, T. S. Selvavinayagam, Karikalan Nagarajan, Kannan Thiruvengadam, Rajendran Krishnan, Priya Rajendran, Paul Kumaran, Makesh Kumar, Padmapriyadarsini Chandrasekeran

**Affiliations:** a ICMR-National Institute for Research in Tuberculosis, Chennai, India.; b Directorate of Medical and Rural Health Services, Tamil Nadu, India.; c Directorate of Public Health and Preventive Medicine, Tamil Nadu, India.

## Abstract

Estimating the burden of TB at the subnational level is critical to planning and prioritizing resources for TB control activities according to the local epidemiological situation. We report the experiences and operational challenges of implementing a TB prevalence survey at the subnational level in India. Information was collected from research reports that gathered data from periodic meetings, informal discussions with study teams, letters of communication, and various site visit reports. During the implementation of the survey, several challenges were encountered, including frequent turnover in human resources, lack of survey participation and community engagement, breakdown of X-ray machines, laboratory issues that delayed sputum sample testing, delays in X-ray reading, and network and Internet connectivity issues that impeded data management. To help ensure the survey was implemented in a timely manner, we developed several solutions, including planning ahead to anticipate challenges, ensuring timely communication, having a high commitment from all stakeholders, having strong team motivation, providing repetitive hands-on training, and involving local leaders to increase community engagement. This experience may help future states and countries that plan to conduct TB prevalence surveys to address these anticipated challenges and develop alternative strategies well in advance.

## Abstract (precis)

TB prevalence surveys can help estimate the burden of disease and prioritize resources for TB control. Anticipating operational challenges and addressing them in a timely manner can help ensure the successful implementation of the survey.

## Teaser

**Key Findings:**

- The reliance on mobile X-ray vans, availability of radiographers to read X-rays, adequate facilities for collecting and transporting sputum, and sufficient laboratories to process the samples posed challenges to implementing the Tamil Nadu TB prevalence survey.
- Issues with Internet connectivity hindered data syncing.
- In some areas, low participation and lack of community engagement posed additional challenges. Adapting the survey to the convenience of the community, involving local leadership in the survey, and providing training on community engagement strategies increased participation.

**Key Implications:**

- Stakeholders can learn from this experience in conducting large-scale TB surveys in endemic settings within the framework of existing systems to plan ahead for potential challenges.
- Strong monitoring by an expert technical group during survey implementation can help identify and resolve operational challenges in a timely manner.

## INTRODUCTION

Tamil Nadu, a state in southern India with a population of 72 million, has a higher prevalence of TB than the national average.^1^ In 2019, the State of Tamil Nadu devised its own strategy for a “TB free Tamil Nadu.”^2^ To monitor TB elimination efforts, a baseline TB prevalence survey—the first such survey conducted in Tamil Nadu—was planned from 2021 to 2022. TB prevalence surveys can provide a reliable measurement of the burden of TB in high-burden settings.^3^ Having various stakeholders involved, a sample size of about 1.14 million, and a geographical area of 130,058 square km, we faced various challenges in implementing the survey.^1^ We describe these operational challenges and the solutions developed to address them and recommend ways to ensure successful implementation in the future.

The health care delivery in Tamil Nadu occurs at 3 levels, which is not exclusively for TB. At the primary level, primary health centers form the backbone of the state’s health system and come under the public health department. Primary health centers serve not only to manage various diseases but also to meet the holistic health care needs of the local population at the peripheral level. At the secondary level, various hospitals deliver health care services to the population and focus more on managing diseases in the hospitals. Almost all districts have a medical college that offers tertiary-level care and serves as a referral center from the primary and secondary levels. The colleges come under the department of medical education.

Routinely, presumptive cases of TB are identified at all 3 levels and are linked to the state TB program, which has a network of TB diagnostic laboratories. The state TB program has a dedicated cadre of staff at each district that is responsible for initiating treatment, ensuring treatment completion, and providing support for TB cases.

## STATE TB PREVALENCE SURVEY

Stakeholders both from and outside the state TB program contributed significantly to implementing the state TB prevalence survey. Though the implementation was planned before the COVID-19 pandemic, the survey was conducted after all lockdown restrictions were removed and after the second wave of the COVID-19 pandemic in Tamil Nadu. We conducted a cross-sectional survey in 143 clusters, with 800 participants in each cluster, covering a population of about 114,000 population across 33 districts in Tamil Nadu. Probability proportionate to size was used to allocate the number of clusters to each district. Village health nurses from the public health department were responsible for community mobilization.

A door-to-door census was conducted within the selected cluster, and all households were visited. All eligible persons aged 15 years and older who stayed in the cluster for more than a month were included. Written informed consent was obtained from eligible participants older than age 18 years and parents/guardians of participants aged 15–18 years. Written assent was also obtained for participants aged 15–18 years.

All eligible participants (except pregnant women) underwent a digital chest X-ray in a mobile X-ray unit. Trained medical officers from the public health department read the X-rays in real time. A radiology panel at the district consisting of 2 independent readers (radiologist/pulmonologist) also read the X-ray, and if there were any discrepancies, a third reader read it to classify it as “normal” or “abnormal.” Medical care at survey sites was provided by doctors of the local primary health center. The local government, in collaboration with the primary health centers of the clusters, was responsible for the safety and needs of the staff.

Survey participants who had symptoms suggestive of TB, a history of TB treatment (previous/current), and an abnormal chest X-ray were eligible for sputum examination. Although the state survey did not have external budgeting for mobile X-ray vans, sputum transport, or testing, these were all provided by the state’s TB control program.

Good-quality sputum was collected from all eligible participants under supervision by trained staff. The first sputum sample was subjected to a cartridge-based nucleic acid amplification test in the nearby testing center to detect TB bacilli and rifampicin resistance. A second sputum sample was collected and transported in the cold chain to a pre-identified quality-assured reference laboratory for smear microscopy and liquid culture. Apart from conducting the TB survey activities, the health camps offered point-of-care blood sugar testing, blood pressure measurement, and body mass index measurements. People with elevated blood sugar levels and high blood pressure were referred to the nearby primary health center for further management. TB cases identified in the survey were referred to the program for treatment initiation and further management.

### Ethical Approval

The study protocol (017/NIRT-IEC/2021) was approved by the Institutional Human Ethics Committee of the National Institute for Research in Tuberculosis.

### Data Collection

The experiences and challenges in implementing the TB prevalence survey were collated into a report that gleaned information from periodic meetings between the central team and site-level staff, letters of communication between various stakeholders, and informal discussions with site staff and collaborators. In addition, details from different departments related to procurements made, services requested, and services monitored were collected. The information was reviewed and broadly organized based on common themes. We describe the challenges in detail and the strategies used to overcome them in the following section.

## OPERATIONAL CHALLENGES AND STRATEGIES TO OVERCOME THEM

### Human Resources Training and Management

The survey involved around 150 staff for this project. Medical officers and pulmonologists were identified and trained at the district level for X-ray reading. There was a high turnover of trained staff as the survey required staff to continuously travel to various places throughout the state. Similarly, many medical officers who were trained were transferred to other places as part of the routine department procedures. Hence, in addition to the refresher training given to the teams at regular intervals, we had to repeatedly provide training whenever a new person joined the team.

To address these challenges, refresher trainings were done in person and through video conferencing at regular intervals to ensure good performance in survey conduction. Assessment modules were used to measure the effectiveness of the repetitive trainings. All staff were aware of all procedures so that even during the COVID-19 crisis, when many staff had fallen sick, the team was competent to manage the survey activities and perform the additional responsibilities for their sick colleagues.

### COVID-19-Related Disruptions

The survey was halted for 2–3 months due to the COVID-19 lockdown. Many staff became sick from COVID-19 infection, and due to the risk of illness, a few were hesitant to rejoin and left the job. Coordinating with the public health department helped mobilize the required personal protective equipment for staff. The national COVID-19 vaccine campaign gave high priority to health care workers, and all survey staff were immediately vaccinated when the vaccine was launched.

Due to the COVID-19 restrictions, there were challenges in the cluster coverage, especially in urban clusters. Many of the households were locked, and many refused to include their household in the study. Participants were hesitant to reveal any symptoms due to fear of being quarantined due to COVID-19. Local community leaders were engaged to combat participants’ fears and concerns, and staff were trained to speak sensitively regarding COVID-19 issues.

### Survey Participation and Community Engagement

In urban areas, there was low participation because many people had to travel to various places for work and were not available during the survey. Some of them had even migrated to their native villages during the COVID-19 pandemic, as they had an option to work remotely. In rural and remote areas, participants had difficulties traveling to the main survey site from their houses. Survey participation increased by extending the hours of activities, providing transport for those participants living far from the survey site, providing refreshments for participants, and offering other health services (e.g., free blood sugar check-ups).

In some clusters, there were issues in which participants from 1 street refused to go to another street where the survey camp was placed. This resulted in changing the survey site many times to enable the participation of all community members. In certain clusters, participants, especially women, were reluctant to participate in the survey without getting concurrence from their husbands and religious heads.

To help increase community engagement, at each cluster, the district TB officer and local community leaders held an inauguration for the survey. In certain clusters, religious leaders were approached to motivate the community to participate. The slow rate of cluster coverage was tackled by having strong team motivation, constant supervision and monitoring at the cluster site by the central team, and repeated trainings on communication and community engagement strategies.

### X-Ray Machine Breakdowns

Having an operational X-ray vehicle was crucial and a rate-limiting factor that required immediate attention. To stay on track with the survey timelines, we had to take a minimum of 100 X-rays each day per team. Due to the long hours needed to conduct the survey, we faced several breakdowns of X-ray machines and generators that were used for the survey. Thus, the survey had to be suspended temporarily until the X-ray unit was repaired. Because the outdoor temperatures were 37–40 degrees Celsius, X-ray machines and generators often overheated despite having an air conditioner in the X-ray van. Backup machines were available when breakdowns occurred. Having a comprehensive maintenance contract with the supplier with regular maintenance reduced the time that equipment could not be used.

### Laboratory Issues

The sputum samples had to be transported and tested through the routine TB program in the state. We faced delays in samples being delivered to the reference laboratories by parcel services, sometimes more than 48 hours. This was a major challenge, especially in the summer, because it was difficult for the cold chain to be maintained, resulting in sample liquefaction and a high risk of contamination. When unavoidable delays in sample transport were anticipated, care was taken to arrange for the storage of samples in nearby hospitals with the cooperation of the local public health department.

In certain clusters, the number of participants eligible for sputum testing by X-ray was more than expected, posing a challenge because of the limited availability of liquid culture tubes in the middle of the survey. Until the supply of tubes was restored, we switched to solid culture for a few weeks to avoid a sample backlog for fear of contamination.

When there was a shortage of consumables, strong leadership among all the stakeholders helped mobilize resources from various sources without affecting the flow of the survey.

### Delays in X-Ray Reading

During the COVID-19 pandemic, district TB officers were assigned COVID-19-related activities, and pulmonologists/radiologists were assigned COVID-19 duties as well as outpatient duties, so it was challenging for them to report X-rays on the same day or the next day. Despite these challenges, we successfully completed all X-ray readings within 48 to 72 hours. Due to the delay in X-ray reporting by a few days, the survey team faced many challenges with sputum collection because some participants had traveled out of town.

To address this challenge, digital X-rays were uploaded to the central server where staff could read the images from any place at any time.

### Data Management

There were network issues during natural calamities, and in some rural areas, the network signal was very poor. The Internet connection with the devices used by enumerators had issues, as the routers in the van had poor signal strength. Hence, staff came to the survey site often to sync data, resulting in delays, especially in rural areas where households were situated far apart. In some rural areas of the state, some team members traveled to nearby places that had good network coverage so that they could sync the data to the central server daily. Daily data cleaning and checking and daily communication with the team ensured high-quality data collection in a timely manner.

## DISCUSSION

TB prevalence surveys are difficult to conduct for a variety of reasons, including the dependence on mobile X-rays, radiographers to read X-rays, facilities for collecting and transporting sputum, and laboratories to process the samples. In contrast, other surveys depend mainly (or only) on the results of questionnaires.^3^^,^^4^ Team spirit and motivation of the survey team are very important when implementing surveys because staff continuously work at survey sites and may get worn out easily. The survey team’s concerns were addressed in the best possible way to ensure a good-quality survey.

The TB prevalence survey conducted in Tamil Nadu was similar to the national survey conducted in India from 2019 to 2022 and experienced some similar operational challenges.^1^ Of note, the national TB survey hired an agency to transport samples in the cold chain, which could not be done for this survey because of budget constraints. The national survey had the capability to do sputum testing by cartridge-based nucleic acid amplification test in the X-ray van, which provided immediate results. However, in this survey, sputum had to be transported to nearby nucleic acid amplification test testing labs for results.

The challenges we experienced during this survey are similar to those experienced by various surveys conducted in other countries. Challenges with X-ray machine breakdowns are one of the major factors stated by various TB surveys across the world.^5^^–^^8^

The challenges with sputum transportation and the potential for cross contamination that we faced have also been reported in TB surveys conducted in Bangladesh, Sudan, and Malawi.^8^ Although we faced some backlogs and delays in culture inoculation because of the high volume of specimens in the reference laboratories, switching to a solid culture medium for a few weeks reduced the backlog until the liquid culture consumables were mobilized.

Many surveys across the world have reported major challenges in X-ray reading.^5^^–^^8^ We faced some challenges in X-ray reporting but managed to have the X-rays read by radiologists/pulmonologists 48–72 hours after the completion of each cluster by enabling readers to access the digital X-rays within hours on the central server.

Electronic data capture systems significantly facilitated and increased the efficiency of data collection, validation, and analysis. Digital data collection and syncing data with the central server ensured data quality assurance as the central team could do quality checks daily. Any issues were rectified the next day by the field team. The data were ready to analyze within the next few days after the completion of site activities.

Based on our experience, we summarize lessons learned and provide recommendations to help ensure the successful implementation of TB prevalence surveys in the Table.

**TABLE.** Lessons Learned and Recommendations to Address Operational Challenges Faced in Conducting TB Prevalence Surveys, Tamil Nadu, India

**Challenges Faced**	**Recommendations**
Human resource training and management	The quality of the entire survey depends on staff training. Staff attrition in large-scale surveys should be anticipated, and ready-made training materials in audio-video format should be available to train staff anytime, anywhere.
COVID-19-related disruptions	Staff safety must be a priority during emergency situations to avoid delays in the survey.
Low survey participation and community engagement	The involvement of community leaders and local community engagement is critical for the survey. Implementing an information, education, and communication program through local health authorities can sensitize the community and increase survey participation.
Frequent X-ray equipment breakdowns	Planning cluster activities in sessions of 3–4 hours each and scheduling a 4-hour break for the X-ray unit reduces the frequency of X-ray breakdowns. Care should be taken to avoid operating the X-ray unit when the outdoor temperature is high.
Laboratory issues	Funding for surveys should try to include a special agency for sputum transportation with cold chain monitoring exclusively for the survey. Reference laboratories must be prepared with adequate staff and consumables to manage increases in samples. Strong leadership and effective collaborative networks should be engaged to mobilize consumables quickly and help manage supply chain issues.
Delays in X-ray reading	An adequate budget should be allocated and a trained standardized radiologist/pulmonologist should be assigned to read X-rays daily so that the eligible participants can be approached as early as possible. Wherever funding is possible, incorporating artificial intelligence/teleradiology should be considered.
Data management and Internet connectivity issues	Having a competent and responsive data management team from survey design to completion is essential. To avoid data collection delays caused by connectivity issues, the survey team should be prepared to collect data on paper. The use of bar codes and bar code scanners reduces errors in data entry and matching records.

## CONCLUSION

This survey serves as a model of different stakeholders collaborating in conducting large-scale surveys in endemic settings. The survey also provided a platform to increase awareness about TB in the community. Strong monitoring provided by an expert technical group was a major strength of the survey that helped identify and resolve problems in a timely manner. The challenges faced and recommendations made can be useful for states and countries intending to conduct a TB prevalence survey to anticipate potential challenges during the survey planning process and devise plans to address them well in advance.

## Notes

**Peer Reviewed**

**First published online:** January 25, 2024.
